# Optimizing schedules for the VLBI global observing system

**DOI:** 10.1007/s00190-019-01340-z

**Published:** 2020-01-08

**Authors:** Matthias Schartner, Johannes Böhm

**Affiliations:** Department of Geodesy and Geoinformation, TU Wien, Wiedner Hauptstraße 8-10, 1040 Vienna, Austria

**Keywords:** Very long baseline interferometry (VLBI), VLBI global observing system (VGOS), VieSched++, Vienna VLBI and satellite software (VieVS), IVS, Scheduling of the VLBI observations

## Abstract

Very long baseline interferometry (VLBI) scheduling is a challenging optimization problem. With the development of the new VLBI global observing system (VGOS) consisting of smaller but very fast slewing antennas, new opportunities arise. In this work, we give a deep insight into optimized VGOS scheduling using a newly developed VLBI scheduling software called VieSched++, and we show how different scheduling parameters and approaches affect the precision of geodetic results. Therefore, the results of over one thousand generated schedules and over one million simulated sessions are analyzed. The simulations reveal that the most important parameters to optimize VGOS schedules with VieSched++ are the so-called weight factors. A proper selection of individually optimized weight factors can improve the quality of a schedule significantly. It is shown that the values of the weight factors used to generate the schedule are highly correlated with the expected precision of the geodetic parameters. We highlight the benefit of selecting schedules based on large-scale Monte Carlo simulations and show why scheduling statistics like the number of observations or the sky-coverage are not necessarily the best metric to evaluate schedules.

## Introduction

The very long baseline interferometry (VLBI) has been developed by radio astronomers in the 1960s and was soon after used for geodesy as well. Today, it plays an important role in the realization of the terrestrial reference frame (Altamimi et al. 2016) and has the unique capability to determine the celestial reference frame at radio frequencies (Fey et al. 2015). Furthermore, it can be used to determine the orientation of the Earth in space, which is essential for today’s positioning and navigation applications, and it is used for all kinds of related research.

The VLBI Global Observing System (VGOS) as the next-generation VLBI system is necessary to reach the goal of the Global Geodetic Observing System (GGOS) (Plag and Pearlman 2009) of providing station coordinates with an accuracy of 1 mm and velocities of 0.1 mm per year (Niell et al. 2005; Petrachenko et al. 2009). Many studies did test different scheduling strategies for VGOS, like a source-based scheduling approach (Sun et al. 2014) or dynamic scheduling (Lovell et al. 2014; Iles et al. 2018). However, up to now, all official VGOS sessions of the International VLBI Service for Geodesy and Astrometry (IVS) (Nothnagel et al. 2017) are scheduled by a classical scheduling software called *sked* (Vandenberg 1999), which is developed and maintained by the NVI group at Goddard Space Flight Center. Improving the scheduling approaches for VGOS remains still an open research topic (Petrachenko et al. 2009; Niell et al. 2018).

Typically, a VLBI schedule is generated scan after scan using a brute force approach of investigating all possible scans at a time (Gipson 2016; Schartner and Böhm 2019). All these scans are evaluated based on different optimization criteria, like the improvement in sky-coverage or the number of observations (Schartner and Böhm 2019). Based on those evaluations, the best possible scan at the investigated time is selected and scheduled and the process is repeated until the full schedule is generated (Gipson 2010). However, many different optimization criteria exist and typically a combination of multiple criteria is used to improve the quality of the schedule. Unfortunately, some of the criteria are competing against each other, like the need to optimize the sky-coverage above all stations and the need to provide a high number of observations (Gipson 2010; Schartner and Böhm 2019). While the first one implies that the station is slewing long distances between scans, the later one tries to minimize the slew time and rather spend more time on observing sources. The difficulty lies in finding a good set of scheduling parameters which balances all needs and lead to the highest possible accuracy for the estimated geodetic parameters during analysis.

**Fig. 1:**
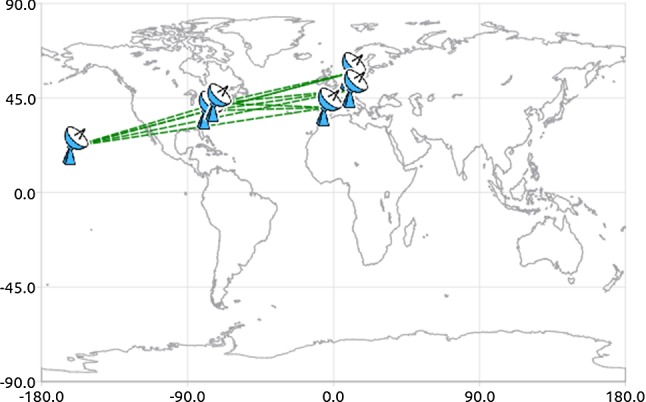
Network geometry of session VT9175

With the recent development of a new VLBI scheduling software called VieSched++ (Schartner and Böhm 2019) which is part of the Vienna VLBI and Satellite Software (VieVS) (Böhm et al. 2018) large-scale simulation studies can be conducted to improve our understanding between VLBI scheduling optimization approaches and geodetic results. For this purpose, the official International VLBI Service for Geodesy and Astrometry (IVS) VGOS session *VT9175* is investigated. VT9175 is the most recent submitted VGOS schedule at the time where this study is made. Figure 1 visualizes the network geometry of this session. It consists of six stations listed in Table 1. The network includes only stations in Europe and North America and can be seen rather as a regional network than a global one. Four of the six stations are fast slewing antennas while the other two, namely GGAO12M and WESTFORD, have significantly lower slew rates which add difficulty in generating an optimized schedule. Moreover, the azimuth span of station WESTFORD is exactly 360$^{\circ }$ and does not include any overlaps as usually common for geodetic VLBI antennas. This adds another difficulty to the scheduling generation since the slewing directions are way more restricted this way.

**Table 1:** Antennas participating in *VT9175* and their key attributes for scheduling

Station	Slew rate az (deg/min)	Slew rate el (deg/min)	Azimuth span (deg)
GGAO12M	300	66	540
KOKEE12M	720	300	500
ONSA13NE	720	360	540
RAEGYEB	720	360	480
WESTFORD	200	120	360
WETTZ13S	720	360	540

In the following work, different scheduling optimization approaches are used to generate an optimized schedule for session *VT9175* based on large-scale Monte Carlo simulations (Metropolis and Ulam 1949). Section 2 discusses the different approaches and parameters used to generate the schedules and how those are further simulated and analyzed. Section 3 shows the expected geodetic results, based on repeatability values of the estimated earth orientation parameters (EOPs) and station coordinates. The repeatability rep of a parameter *v* is defined as the standard deviation over the estimated parameter values over the individual simulations *i*

1 $$\begin{aligned} \text {rep}_v = \sqrt{\frac{1}{N-1} \left( \sum \nolimits _{i=1}^N v_i - {\bar{v}}\right) ^2} \end{aligned}$$

with the total number of simulations *N* and ${\bar{v}}$ being the mean value of *v*. It compares the results gained from the different scheduling optimization approaches, like introducing a minimum slew distance 3.1, the sky-coverage definition 3.2 and the impact of changing weight factors 3.3. Section 4 presents conclusions.

## Method

The method used to investigate the research goal is based on large-scale Monte Carlo simulations. All steps are performed using well-tested state-of-the-art software developed at TU Wien. For the generation of the scheduling, VieSched++ is used. In total over 7000 schedules are generated. Every schedule is then simulated and analyzed 1000 times using VieVS leading to a total of over seven million simulations. Based on those simulations, repeatability values are calculated. The schedules are compared based on these repeatability values to determine the best schedule.

### Scheduling

The VieSched++ schedule is optimized using the multi-scheduling feature (Schartner and Böhm 2019, 2019). Similar to the officially observed VGOS schedules, the scan duration is fixed to 30 s for all stations. However, the slew duration is not fixed to 30 s and is instead calculated based on the individual slew rates of the stations provided in the official sked-catalogs (Vandenberg 1997). The observed VT9175 schedule uses a minimum slew duration of 30 s, which is necessary due to hardware restrictions for writing the data to disk. Since this very high minimum slew duration heavily diminishes the benefit of having fast slewing VGOS antennas and because we believe that this restriction will vanish in future, this constraint is not applied in this study. Finally, the same source list is used as in the official IVS VGOS schedule and all other restrictions, such as horizon masks are applied to generate valid schedules.

The VieSched++ multi-scheduling feature is used to vary three weight factors, the sky-coverage definition, and the minimum slew distances. Additionally, it is investigated whether subnetting should be considered or not. Subnetting is a technique which allows the scheduling software to investigate two scans at the same time. Since typically a VLBI network spans the whole world and a schedule is generated scan after scan this is critical since the visible sky above each station is different and no source can be seen simultaneously from all over the Earth. Although the investigated network consists of only six stations, early simulations show, that subnetting is beneficial for the geodetic solution, and all schedules discussed in this paper are made with considering subnetting.

The three weight factors tested refer to the duration of a scan, the number of observations per scan and the improvement in sky-coverage per scan. The tested values are $\{0,0.33,0.67,1\}$. The idle time weight factor was either fixed to 0.5 or 1.0 with an interval of 300 s. More information about weight factors in general and how they affect the scheduling process can be found in Schartner et al. (2017) and in more detail in Schartner and Böhm (2019). The reason why only two different values for the idle time weight factor are tested is because VGOS schedules are expected to observe a scan every minute or even more often. In consequence, long idle times should not occur, and therefore, changes on the idle time weight factor should not influence the result too much. The purpose of the idle time weight factor is to provide a safety net to force the scheduling of stations which are not included in the last couple of scans to make sure to avoid long idle times at the stations.

The sky-coverage definition was tested with an influence distance of $\{15,30,45\}$ degrees and an influence time of $\{900,1800,3600\}$ seconds. Half cosine functions are used as transfer functions to calculate the saturation of the sky-coverage based on the angular distance and the time distance between observations (Schartner and Böhm 2019).

Schedules with the combination of all these parameters are generated and compared. This leads to a total of 1152 schedules, three weight factors with 4 values, one weight factor with two values and two sky-coverage parameters with three values, thus $4\times 4\times 4\times 2\times 3\times 3 = 1152$ Additionally, it is investigated whether the introduction of a minimum slew distance is beneficial. Therefore, schedules with a forced minimum slew distance are generated with an angular distance of $\{0,10,20,30,40,50,60\}$ degrees.

### Simulation

As already discussed in Sect. 2, every schedule is simulated 1000 times using VieVS leading to a total of over several million simulations. The simulations include tropospheric time delays, clock drifts, and white noise (Pany et al. 2011). For the troposphere, all stations are simulated using a tropospheric refractive index structure constant $C_n$ of ${2.0\times 10^{-7}}$ (m$^{-1/3}$) up to 2000 m (Nilsson et al. 2007) and a wind velocity of 8 m/s toward east. The clock is simulated as the sum of random walk and integrated random walk with an Allan Standard Deviation (ASD) of ${1\times 10^{-14}}$ s after 50 min (Herring et al. 1990). Additionally, 4 picoseconds of white noise are added to the observations similar as shown in Petrachenko et al. (2009) for earlier VGOS simulation studies.

As it is the case in previous studies, we do not simulate source structure effects in this work, mostly due to limitations in the software packages, although the effect of source structure on geodetic VLBI is highly significant (Anderson and Xu 2018). Adding source structure simulations into the process chain will be part of future studies.

### Analysis

The analysis of the simulated sessions is done in VieVS. The troposphere is estimated using piecewise linear offsets with an interval of 15 min. Tropospheric gradients in north and east direction are estimated every 30 min. Additionally, loose constraints are introduced between the estimates. The EOPs are estimated as very tightly constrained daily piecewise linear offsets, which corresponds to an estimate of one offset per session. The station coordinates are estimated as one offset per session and the clock is estimated as a rate, a quadratic term, and piecewise linear offsets every 60 min. No source coordinates are estimated in this study.

Instead of using the formal uncertainty information of the estimated geodetic results provided by the least-squares method, repeatability values are calculated from the 1000 individual solutions per schedule. The drawback of using repeatability values is, that a relatively high number of simulations per schedule are necessary. These repeatability values are used to compare the different schedules against each other. However, using the formal uncertainties instead of repeatabilities would lead to the same conclusions discussed in Sect. 3.

**Fig. 2:**
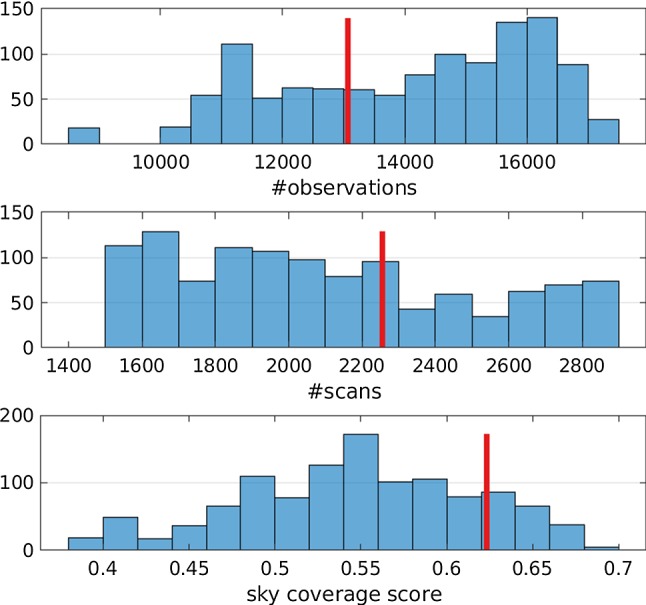
Histogram of scheduling statistics for all generated schedules. The selected schedule is highlighted in red. The sky-coverage score is a metric to compare the sky-coverages of different schedules. It is ranging from 0 (poor sky-coverage) to 1 (perfect sky-coverage)

## Results

The scheduling and simulations are done iteratively to test different parameters and approaches. Here the results of the last iteration are discussed. This iteration included a total of 1152 different schedules derived from different weight factor values and sky-coverage definitions serving as scheduling input parameters. Figure 2 depicts the number of observations, number of scans and average sky-coverage score. In the background, a histogram of the results of all 1152 schedules is visualized. The red line marks the value of the “selected” schedule. The selected schedule is chosen based on the repeatability values of the average 3D station coordinate repeatability, defined as $\sqrt{x^2+ y^2+ z^2}$, and the repeatability of the five EOP estimates. The 1152 different solutions span the range of the repeatability values per parameter. The selected schedule provides the best overall performance based on the six parameters relative to the range of the repeatability values. In practice, it is realized as the schedule whose repeatability values are below a minimum quantile for all six parameters. Table 2 lists the key scheduling parameters used to generate the selected schedule. The selected schedule is the schedule which would be sent to the stations as the official schedule. Figure 2 reveals that the number of observations of the schedules generated with VieSched++ varies mostly between 10,000 and 17,000 and the number of scans varies between 1400 and 2900. Additionally, Fig. 2 depicts the sky-coverage score calculated in VieSched++. While during the scheduling process, the sky-coverage saturation is defined through an influence distance, an influence time and two transfer functions, additional sky-coverage scores are calculated at the end of the scheduling process to provide a metric which can be used to compare the sky-coverage between different stations and schedules. The sky-coverage score displayed in Fig. 2 is calculated using the following procedure: First, the sky above the station is distributed in 37 areas of approximately equal size. This is done in two ways, once by using five elevation layers and once using four elevation layers. The two different distribution approaches are done to avoid possible clustering of observations at the edges of the areas. Every 30 min, with an overlap of 15 min, the number of areas in which observations are scheduled is counted and divided by the total number of areas—in this case 37. This is done for both area distribution approaches and the average of the two resulting values is used as the sky-coverage score within the tested time interval. Based on this approach, the sky-coverage is considered perfect for the tested time period if observations are scheduled in a way that they cover all 2 times 37 areas within the time period. The station sky-coverage score is defined as the average of the sky-coverage scores over all time intervals. The average over all station sky-coverage scores is the final sky-coverage score of this session, listed in Fig. 2.

**Table 2:** Key scheduling parameters used to generate the optimized schedule based on the results from the repeatabilities

Parameter	Value	Unit
Sky-coverage weight factor	0.67	
Number of obs/scan weight factor	0.33	
Duration weight factor	0.67	
Idle time weight factor	1.00	
Sky-coverage influence distance	45	(deg)
Sky-coverage influence time	1800	(s)
Sky-coverage transfer functions	Half cosine	
Minimum slew distance	0	(deg)
Considering subnetting	Yes	
Minimum scans per source	3	

**Fig. 3:**
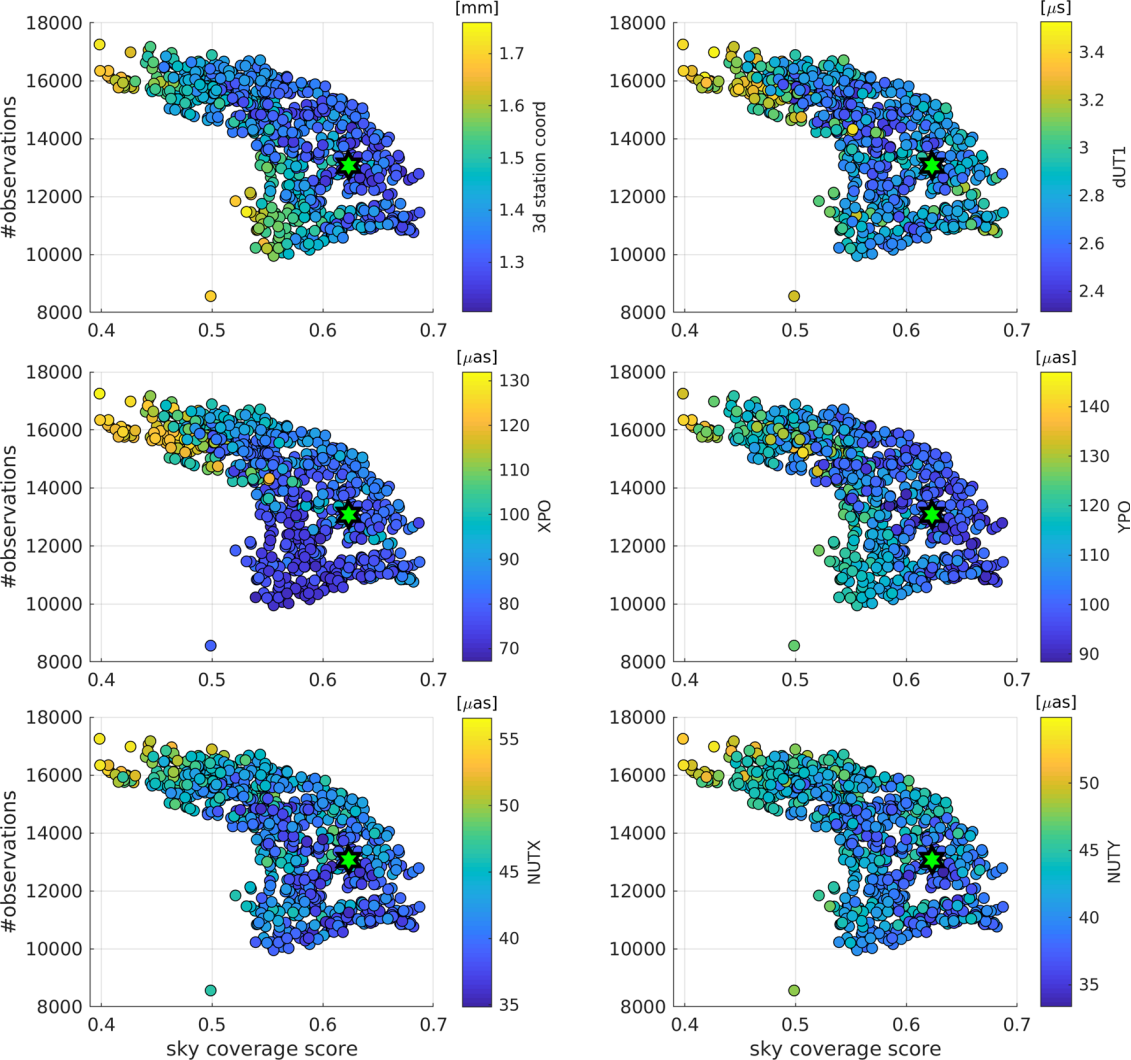
Number of observations versus sky-coverage score with color-coded repeatability values of the average 3D station coordinates and EOP parameters

When looking at the sky-coverage score of the selected schedule, it can be seen that it has a relatively high sky-coverage score. Therefore, based on the simulations and based on the approach of generating over one thousand different schedules per session and comparing these schedules based on the repeatability of the five EOP and the 3D station coordinates from one thousand simulations per schedule, a schedule was selected as the best one which focuses more on having a good sky-coverage than on providing a high number of observations. Figure 3 plots these two values against each other for the generated schedules. The distribution of the markers visualized in Fig. 3 shows that a high sky-coverage score leads to a lower number of observations and vice versa, as discussed in Sect. 1. Additionally, Fig. 3 highlights another problem in selecting a proper schedule for a geodetic session. The estimated repeatability values of the different solutions are color-coded for the average 3D station coordinates and the EOP parameters. It can be seen that different areas provide the highest precision for the different parameters. If the scientific goal of the session is not defined through one single parameter, like dUT1 for intensive sessions or station coordinates for terrestrial reference sessions, it is difficult to decide which schedule is the best one for this session.

**Fig. 4:**
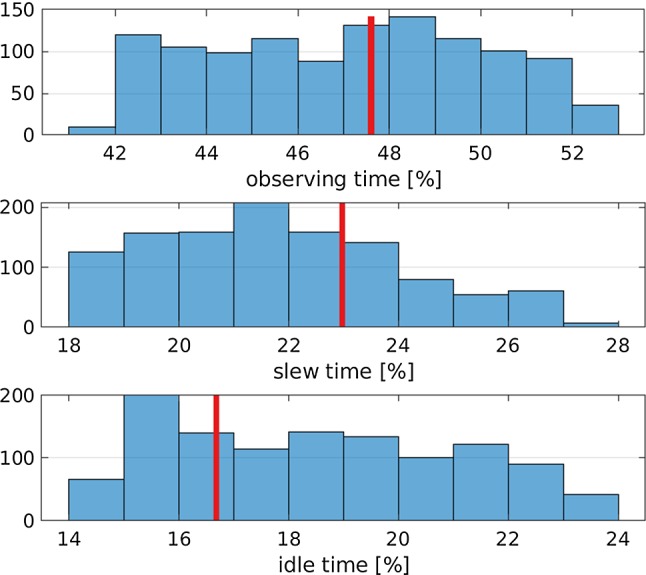
Histogram of the time spent during the VGOS sessions for all generated schedules. The selected schedule is highlighted in red

**Fig. 5:**
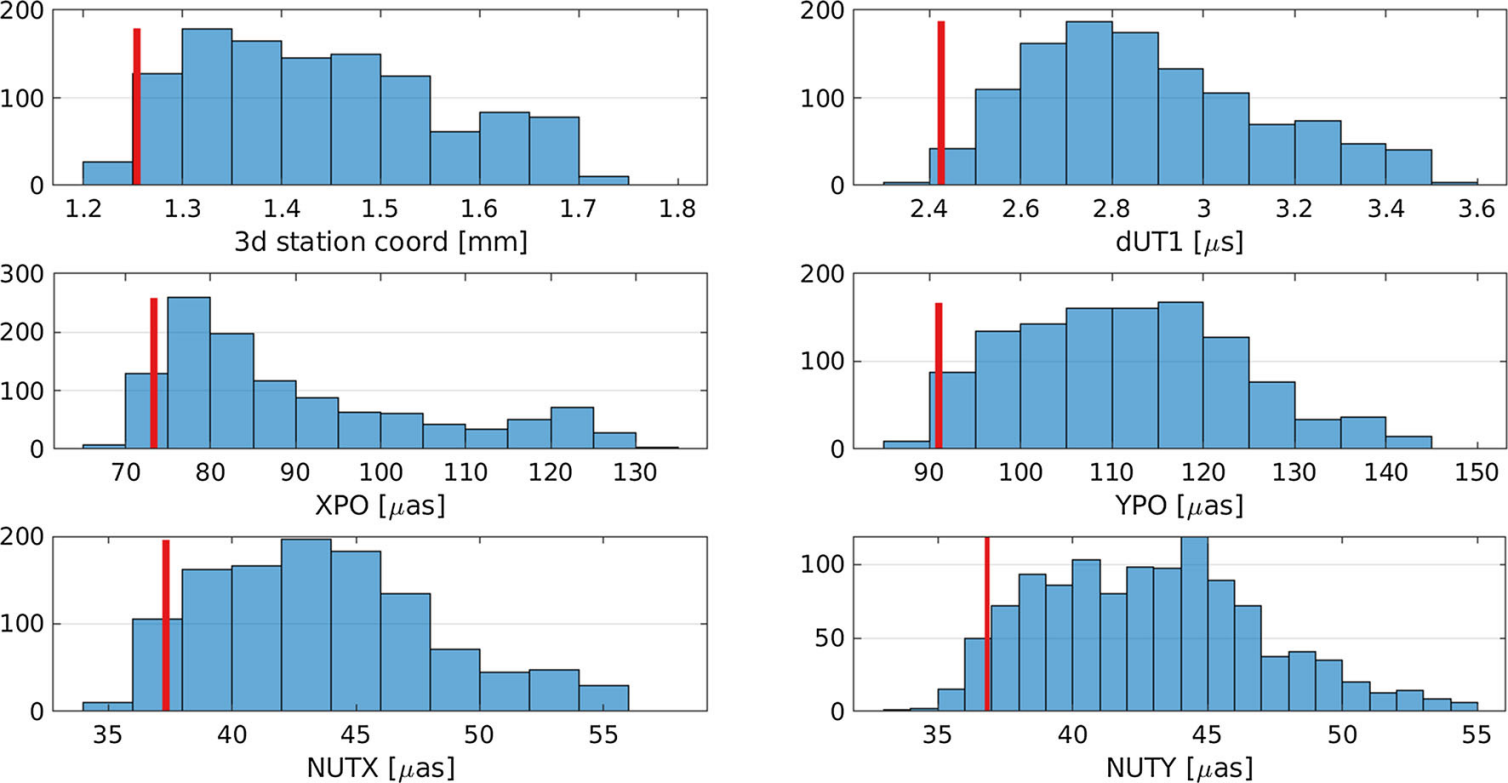
Histogram of the estimated repeatabilities for geodetic parameters based on Monte Carlo simulations. The selected schedule is highlighted in red

This explains why the selected schedule, which was chosen through the average repeatability values of the simulated geodetic results, is located in the middle. Surprisingly, the best schedule is neither especially good in terms of the number of observations nor sky-coverage score. There are other candidates who are performing better based on these two metrics. This means that neither the number of observations nor the sky-coverage distribution alone is a perfect candidate to compare schedules for geodetic purposes. Instead, it is critical to simulate the schedules and draw a conclusion based on the simulated geodetic results as done in this study. However, both the number of observations and the sky-coverage can be used as a first guess.

For clarification purposes, it should be noted here that all schedules are generated by making the best possible schedule within the rules of the scheduling logic defined through the scheduling input parameters, meaning none of the schedules are made poorly on purpose. However, the estimated repeatability values vary greatly, between 1.8 and 1.2 mm for the station coordinates and 130–70 $\upmu$as for polar motion in *x* direction. Therefore, the precision can be improved by $\approx 40$% by simply changing the scheduling parameters. This highlights that it is necessary to understand how the scheduling parameter affects the geodetic results which is one of the main motivations of this study.

Figure 4 shows the percentages of time spent during the session rationed in observing time, slew time and idle time. This does not include calibration time and time for the field system commands as they are fixed per scan. The stations are observing for almost half the session time, while they spend roughly one quarter in slewing. The high observing time can be explained by the fixed scan time of 30 s. In the future, the observing time will be reduced significantly by increasing the recording rate of the observations and improving the hardware setup at the stations. The original VGOS plan was to provide observations as short as 5 s (Petrachenko et al. 2009), which would provide more time for additional observations. To reduce the idle time, the leftover idle time could then be used as an additional observing time to increase the signal to noise ratio. However, this would increase the amount of recorded data. Since the data transfer and correlation is considered a bottleneck of today’s VGOS observations, this option is more of theoretical interest and not considered in this study.

Figure 5 depicts the expected EOP and 3D station coordinate precision based on the repeatability values of the 1000 simulations. The selected schedule is never the best performing for any of the parameters; however, it is selected in a way to yield good results throughout all parameters. If the main interest of the session would be only to provide high-quality station coordinates, the selection of the schedule can be adjusted accordingly.

### Minimum slew distance

**Fig. 6:**
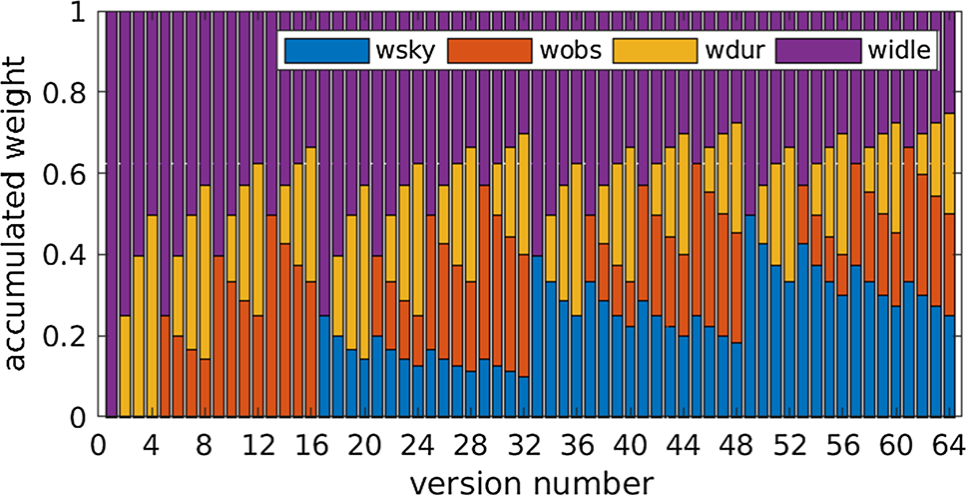
Ratio of weight factors used to generate the schedules

While adjusting the weight factors is the recommended way of optimizing the schedule (Schartner et al. 2017; Schartner and Böhm 2019), another approach is to introduce a minimum slew distance, meaning that the telescopes have to slew at least this angular distance between two scans. To test this approach, minimum slew distances between 0$^{\circ }$ and 60$^{\circ }$ are introduced in 10-degree steps and schedules are generated based on these restrictions. For every minimum slew distance restriction, 64 schedules are generated varying the weight factors as described in Sect. 2.1 with a fixed idle time weight of 1.0. This is done to make sure to generate an optimized schedule in all cases and to minimize the effect caused by other scheduling parameters. Together, this leads to a total of 448 investigated schedules. Since only the relative ratio of the weight factors is important the weight factor influence can be shown as in Fig. 6. As already discussed in Sect. 2.1, the idle time weight factor is only a safety net making sure that all stations are observing regularly, the relative ratio between the remaining three weight factors is the dominating factor.

**Fig. 7:**
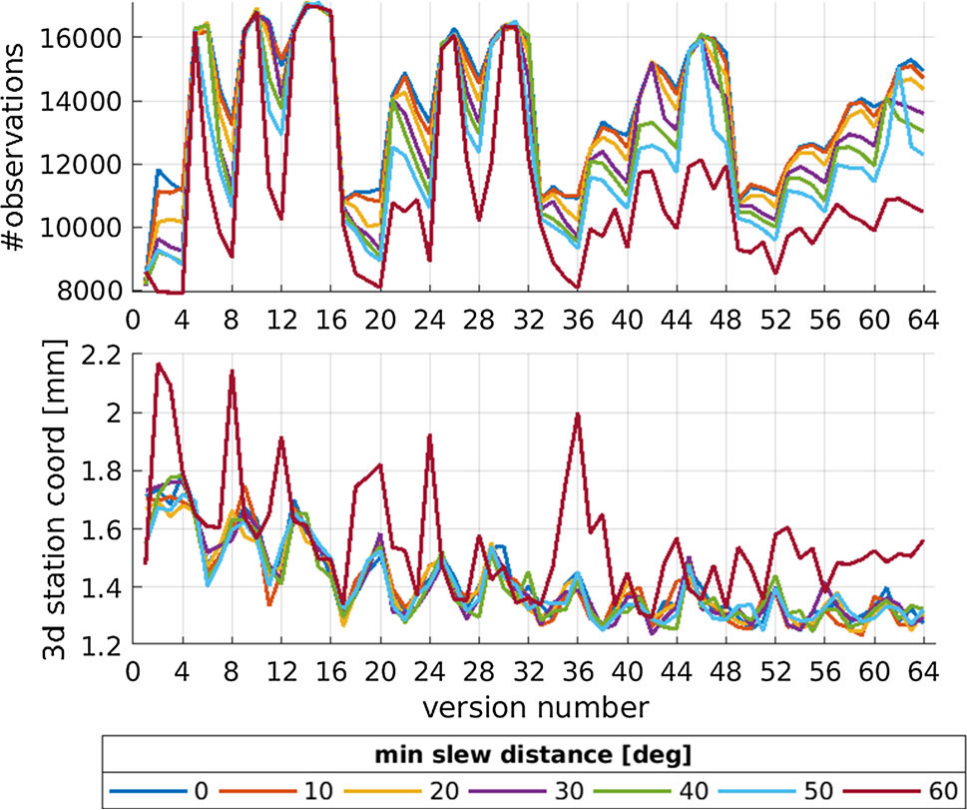
Impact of different minimum slew distances on geodetic results. The abscissa is the version number of the corresponding weight factor ratio shown in Fig. 6

Figure 7 shows the number of observations as well as the expected average 3D station coordinate precision based on the repeatability values of the 1000 simulations per schedule. The abscissa lists the different weight factor versions visualized in Fig. 6, while the effect of different minimum slew distances is shown as color-coded lines. When looking at the number of observations, it can be seen that the higher the minimum slew distance is, the fewer observations are scheduled. This makes sense since the stations are forced to slew longer distances and thus spend longer times slewing which reduces the available time for observations. While the effect is negligible between zero and 20$^{\circ }$ minimum slew distance especially when introducing a 60-degree minimum slew distance the number of observations drops significantly. When looking at the average 3D station coordinate repeatability values neither a positive nor negative effect based on the introduction of a minimum slew distance can be seen up to 60$^{\circ }$. Only the 60-degree minimum slew distance results in higher average 3D station coordinate repeatability values indicating a poorer scheduling result. Therefore, it can be concluded that introducing a minimum slew distance between two subsequent scans does not help to generate a better schedule; in fact, it can diminish the scheduling quality if the value is set too high. While here only repeatability values for the average 3D station coordinates are shown, the same effects can be seen for the EOP repeatability as well as for the formal uncertainties.

When looking at the number of observations and the average 3D station coordinate repeatability in Fig. 7, a clear zigzag pattern can be seen. This pattern corresponds to the different weight factor ratios visualized in Fig. 6. This effect is further discussed in Sect. 3.3.

### Sky-coverage definition

Since the troposphere is considered one of the major error sources in VGOS observations (Petrachenko et al. 2009; Pany et al. 2011; Niell et al. 2018) and the tropospheric zenith wet delay is highly correlated with clock parameters as well as the station height (Nothnagel et al. 2002), it is necessary to schedule observations at different azimuths and elevations thus leading to a good sky-coverage. The definition and implementation of this requirement in scheduling software are not straightforward. Different approaches can be used, like distributing the sky in different areas (Sun 2013). VieSched++ calculates the sky-coverage saturation for every possible observation based on the angular distance and time between the new observation and all previously scheduled observations (Schartner and Böhm 2019).

**Fig. 8:**
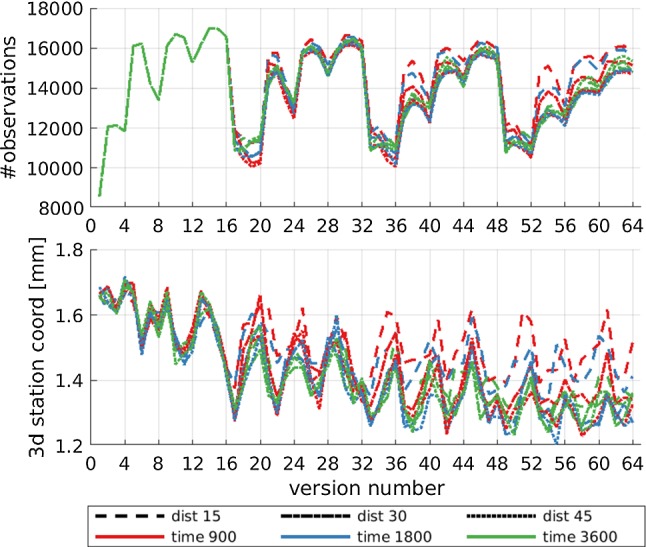
Impact of different sky-coverage definition on geodetic results. The abscissa is the version number of the corresponding weight factor ratio shown in Fig. 6

Figure 8 shows the effect of different sky-coverage definitions. Similar to Fig. 7 the abscissa lists different weight factor ratios visualized in Fig. 6. The line style is used to distinguish different angular influence distances, while different influence times are color-coded. In total, combinations of three different distances and three different times are tested, leading to nine solutions. When looking at the number of scheduled observations in Fig. 8, no big differences can be seen for most versions between the different sky-coverage definitions. Moreover, the first 16 schedules are completely identical. This can be explained by looking at the weight factor ratio in Fig. 6. The first 16 schedules have zero weight on optimizing the sky-coverage. Therefore, the nine different sky-coverage definitions do not influence the scheduling process, leading to identical schedules. Versions in Fig. 8 with high weight on the sky-coverage weight factor show the biggest differences between the different sky-coverage definitions.

The agreement of the repeatability values from the different sky-coverage definitions for the first 16 versions can be used to validate the simulation approach. Since the schedules are identical one would expect identical repeatability values. Figure 8 depicts the repeatability values of the average 3D station coordinates. The results agree quite well with each other (e.g., low scatter between the 9 lines for the first 16 versions). The same is true for all EOP. Therefore, it is safe to conclude that the solutions are trustworthy and the repeatability values of the 1000 simulations can be safely used as a metric for comparisons.

Although the differences based on the number of observations do not look significant, the differences based on the average 3D station coordinate repeatability are more prominent. In particular, when using only an angular influence distance of 15$^{\circ }$, shown with a dashed line, the repeatability values are higher. This indicates that 15$^{\circ }$ influence distance is not enough for VGOS sessions. Differences between the 30- and 45-degree influence distance and between the influence times cannot be seen clearly.

As discussed at the end of Sect. 3.1, a clear zigzag pattern is evident between the different versions, which can be explained through the impact of the weight factors and is further discussed in Sect. 3.3.

### Impact of weight factors

Weight factors are used to define how much certain optimization conditions contribute to the scan selection during the scheduling generation procedure (Schartner and Böhm 2019). Since they directly determine which scan is scheduled, great care must be taken to choose good weight factors for the given network geometry and scientific purpose.

**Fig. 9:**
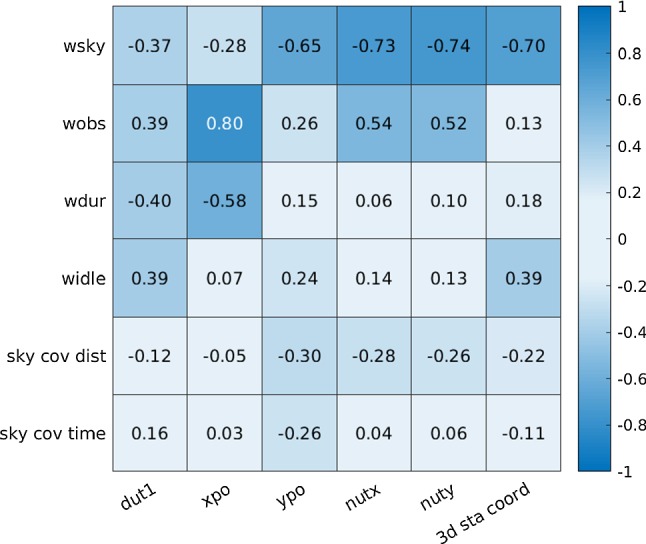
Correlations between scheduling parameters including weight factors and sky-coverage definitions with geodetic results

When looking at Figs. 7 and 8 , the scatter over the different weight factor ratios (abscissa) is higher than the scatter between the tested parameters (lines). This indicates that the selection of good weight factors is still the most important factor for optimizing a VGOS schedule. It can further be seen that the average 3D station coordinate repeatability tends to decrease with a higher version number. Since the relative weight of the sky-coverage factor tends to increase with higher version number, it can be concluded that the sky-coverage weight factor plays the most important role for the 3D station coordinate repeatability. This can be explained by the simulation parameters which were used for simulating the sessions described in Sect. 2.2. The troposphere is clearly the dominant error source in the simulations and the sky-coverage weight factor is designed to help to estimate tropospheric parameters. To further confirm this statement, correlations between scheduling parameters and the estimated repeatability values are calculated and displayed in Fig. 9. In total, six different scheduling input parameters are tested, four weight factors and the sky-coverage definitions. A correlation of zero would mean, that the estimated geodetic result is completely independent of the input of the scheduling parameter. A correlation of plus or minus one would mean that the estimated geodetic result is completely dependent on the scheduling parameter. It can be seen that for the 3D station coordinate repeatability, the sky-coverage weight factor shows the highest correlation, which confirms that it is the dominant scheduling input factor for this parameter. This is also proven by the negative correlations of the sky-coverage weight factor values with the repeatability values. A high weight on this weight factor leads to low values for the repeatabilities, i.e., to good precision. However, it is to note that for the weight factors, only the relative ratio of the weight factor values is of importance and used (Schartner and Böhm 2019), meaning that a high weight on one parameter implicitly leads to low values for the other weight factors. This might explain the positive correlations of some weight factors with the repeatabilities in case of strong negative correlations between a weight factor and the repeatabilities. Additionally, it is to note here that the weight factors are somewhat arbitrary quantities that are used during the scan-by-scan-based generation of the schedule to evaluate which scan is scheduled at which point in time. Moreover, they interact with each other and can not be translated one-to-one with the scheduling outcome, e.g., the total number of observations in a schedule is not only determined by the number of observations per scan weight factor. It is also heavily influenced by the scan duration weight factor.

In general, the correlations of the weight factors are higher than the correlations of the sky-coverage definitions proofing that a proper selection of weight factors is the most important step for generating an optimized schedule. The sky-coverage weight factor together with the number of observations weight factor tends to be the most important one for the tested network.

## Conclusion

In this work, we have given an in-depth analysis of how the scheduling parameters affect the geodetic results of a VGOS session. Therefore, we have generated thousands of schedules and analyzed millions of simulations. We showed, that a proper selection of the weight factors is the dominant factor to generate an optimized schedule. Although we only used session VT9175 to verify this statement in this work, it is true for every VLBI experiment in general. The reason for this is, that the weight factors directly determine which scans are scheduled while many other parameters, such as minimum slew distances, only quantify which scans are possible candidates of being scheduled. For the tested setup, the sky-coverage weight factor plays the most important role; however, other factors play in as well. We have also shown that, while being very important, an optimized sky-coverage alone does not lead to a good geodetic schedule and neither does a high number of observations. Additionally, we highlighted the benefit of evaluating schedules based on large-scale Monte Carlo simulations instead of scheduling parameters like the number of observations or sky-coverage.

We are aware of the fact, that this work is fully based on simulations and that errors in the simulation procedure or especially inconsistencies and limitations of the simulated troposphere turbulence model can heavily affect the results. Therefore, improvements and evaluations of the troposphere turbulence model should be part of future research. Additionally, special focus should be put on properly simulating source structure effects in further work.

The results gained in this study should not be easily applied to other use cases. This is especially true for Table 2 which lists the optimal scheduling parameters found for this session. In operational VGOS scheduling, it is not necessary to generate over one thousand schedules per session. Instead, the results and best parameters found in this study can be used as a starting point for smaller scheduling optimization and should serve as a reference for related research. If the VGOS network changes or if the scan times are adjusted the optimal scheduling parameters can change as well. In particular, our results cannot be translated directly to legacy SX-sessions including slow slewing stations since their scheduling requirements are different from VGOS sessions.

Our final advice is to do this research for every submitted schedule individually to fully optimize every schedule. In particular, we do not recommend to use the same scheduling parameters for every session especially if the network geometry changes. Since a schedule is typically generated scan after scan fluctuations in the scheduling quality can always arise. Therefore, it is beneficial to generate multiple schedules (e.g., 50–100) for one session and compare the results. Fortunately, this work is highly automated in VieSched++ and VieVS making this process very efficient, e.g., scheduling 100 VGOS sessions with a fixed observing duration of 30 s and simulating each session 1000 times takes less than 30 min on a desktop computer with AMD RYZEN 7 2700X processor.

## Data Availability

All simulation results are available upon request and the software packages which are necessary to reproduce the results are open access and can be downloaded from https://github.com/TUW-VieVS/.

## References

[CR1] Altamimi Z, Rebischung P, Métivier L, Collilieux X (2016). ITRF2014: a new release of the International Terrestrial Reference Frame modeling nonlinear station motions. J Geophys Res Solid Earth.

[CR2] Anderson JM, Xu MH (2018). Source structure and measurement noise are as important as all other residual sources in geodetic VLBI combined. J Geophys Res Solid Earth.

[CR3] Böhm J, Böhm S, Boisits J, Girdiuk A, Gruber J, Hellerschmied A, Krásná H, Landskron D, Madzak M, Mayer D, McCallum J, McCallum L, Schartner M, Teke K (2018). Vienna VLBI and satellite software (VieVS) for geodesy and astrometry. Publ Astron Soc Pac.

[CR4] Fey AL, Gordon D, Jacobs CS, Ma C, Gaume RA, Arias EF, Bianco G, Boboltz DA, Böckmann S, Bolotin S, Charlot P, Collioud A, Engelhardt G, Gipson J, Gontier AM, Heinkelmann R, Kurdubov S, Lambert S, Lytvyn S, MacMillan DS, Malkin Z, Nothnagel A, Ojha R, Skurikhina E, Sokolova J, Souchay J, Sovers OJ, Tesmer V, Titov O, Wang G, Zharov V (2015). The second realization of the international celestial reference frame by very long baseline interferometry. Astron J.

[CR5] Gipson J (2010) An introduction to Sked. In: Navarro R, Rogstad S, Goodhart CE, Sigman E, Soriano M, Wang D, White LA, Jacobs CS (eds) International VLBI service for geodesy and astrometry 2010 general meeting proceedings, pp 77–84. https://ivscc.gsfc.nasa.gov/publications/gm2010/IVS-2010-General-Meeting-Proceedings.pdf

[CR6] Gipson J (2016). Sked VLBI scheduling software.

[CR7] Herring TA, Davis JL, Shapiro II (1990). Geodesy by radio interferometry: the application of Kalman filtering to the analysis of very long baseline interferometry data. J Geophys Res Solid Earth.

[CR8] Iles EJ, McCallum L, Lovell JEJ, McCallum JN (2018). Automated and dynamic scheduling for geodetic VLBI—a simulation study for AuScope and global networks. Adv Space Res.

[CR9] Lovell JEJ, McCallum JN, Shabala S, Plank L, Böhm J, Mayer D, Sun J (2014) Dynamic observing in the VGOS era. In: Behrend D, Baver KD, Armstrong KL (eds) International VLBI service for geodesy and astrometry 2014 general meeting proceedings, pp 43–47

[CR10] Metropolis N, Ulam S (1949). The monte carlo method. J Am Stat Assoc.

[CR11] Niell A, Whithney A, Petrachenko B, Schlüter W, Vandenberg N, Hase H, Koyama Y, Ma C, Schuh H, Tuccari G (2005) VLBI2010: current and future requirements for geodetic VLBI systems. Technical Report

[CR12] Niell A, Barrett J, Burns A, Cappallo R, Corey B, Derome M, Eckert C, Elosegui P, McWhirter R, Poirier M, Rajagopalan G, Rogers A, Ruszczyk C, SooHoo J, Titus M, Whitney A, Behrend D, Bolotin S, Gipson J, Petrachenko B (2018). Demonstration of a broadband very long baseline interferometer system: a new instrument for high-precision space geodesy. Radio Sci.

[CR13] Nilsson T, Haas R, Elgered G (2007) Simulations of atmospheric path delays using turbulence models. In: Böhm J, Pany A, Schuh H (eds) Proceedings of the 18th European VLBI for geodesy and astrometry work meeting, pp 175–180

[CR14] Nothnagel A, Vennebusch M, Campbell J (2002) On correlations between parameters in geodetic VLBI data analysis. In: International VLBI service for geodesy and astrometry 2002 general meeting proceedings, pp 260–264

[CR15] Nothnagel A, Artz T, Behrend D, Malkin Z (2017). International VLBI service for geodesy and astrometry. J Geodesy.

[CR16] Pany A, Böhm J, MacMillan D, Schuh H, Nilsson T, Wresnik J (2011). Monte carlo simulations of the impact of troposphere, clock and measurement errors on the repeatability of VLBI positions. J Geodesy.

[CR17] Petrachenko B, Niell A, Behrend D, Corey B, Böhm J, Charlot P, Collioud A, Gipson J, Haas R, Hobiger T, Koyama Y, MacMillan D, Malkin Z, Nilsson T, Pany A, Tuccari G, Whitney A, Wresnik J (2009) Design aspects of the VLBI2010 system. Progress report of the IVS VLBI2010 Committee, June 2009. https://hal.archives-ouvertes.fr/hal-00582342, NASA/TM-2009-214180, 2009

[CR18] Plag HP, Pearlman M (2009). Global geodetic observing system: meeting the requirements of a global society on a changing planet in 2020.

[CR19] Schartner M, Böhm J (2019) VieSched++: a new scheduling tool in VieVS. In: International VLBI service for geodesy and astrometry 2018 general meeting proceedings. https://ivscc.gsfc.nasa.gov/publications/gm2018/16_schartner_boehm.pdf

[CR20] Schartner M, Böhm J (2019). VieSched++: a new VLBI scheduling software for geodesy and astrometry. Publ Astron Soc Pac.

[CR21] Schartner M, Böhm J, Mayer D, McCallum L, Hellerschmied A (2017) Recent developments in scheduling with VieVS. In: Proceedings of the 23rd European VLBI group for geodesy and astrometry working meeting, pp 113–116. http://www.oan.es/raege/evga2015/EVGA2015_proceedings.pdf

[CR22] Sun J (2013) VLBI scheduling strategies with respect to VLBI2010. Geowissenschaftliche Mitteilungen 92(92). http://ub.tuwien.ac.at/diss/AC07814953.pdf

[CR23] Sun J, Böhm J, Nilsson T, Krásná H, Böhm S, Schuh H (2014). New VLBI2010 scheduling strategies and implications on the terrestrial reference frames. J Geodesy.

[CR24] Vandenberg N (1997). Sked’s catalogs program reference manual.

[CR25] Vandenberg N (1999). Sked: interactive/automatic scheduling program.

